# D-K_6_L_9_ Peptide Combination with IL-12 Inhibits the Recurrence of Tumors in Mice

**DOI:** 10.1007/s00005-014-0268-z

**Published:** 2014-02-02

**Authors:** Tomasz Cichoń, Ryszard Smolarczyk, Sybilla Matuszczak, Magdalena Barczyk, Magdalena Jarosz, Stanisław Szala

**Affiliations:** Center for Translational Research and Molecular Biology of Cancer, Maria Skłodowska-Curie Memorial Cancer Center and Institute of Oncology, Gliwice Branch, Wybrzeże Armii Krajowej 15, 44-101 Gliwice, Poland

**Keywords:** Peptide D-K_6_L_9_, Peptide BP1, Glycyrrhizin, IL-12, Anticancer therapy

## Abstract

D-K_6_L_9_ peptide is bound by phosphatidylserine and induces necrosis in cancer cells. In our therapeutic experience, this peptide, when administered directly into B16-F10 murine melanoma tumors, inhibited their growth. Cessation of therapy results, however, in tumor relapse. We aimed at developing a combined therapy involving D-K_6_L_9_ and additional factors that would yield complete elimination of tumor cells in experimental animals. To this purpose, we employed glycyrrhizin, an inhibitor of HMGB1 protein, BP1 peptide and interleukin (IL)-12. Glycyrrhizin or BP1, when combined with D-K_6_L_9_, inhibits growth of primary tumors only during the period of their administration. A long-term tumor growth inhibitory effect was obtained only in combining D-K_6_L_9_ with IL-12. At 2 months following therapy cessation, 60 % of animals were alive. Prolonged survival was noted in mice bearing B16-F10 tumors as well as in mice bearing C26 colon carcinoma tumors.

## Introduction

Peptides used in anticancer therapy kill cancer cells by destroying their cell or mitochondrial membranes. D-K_6_L_9_ is an example of cell membrane-acting peptide which triggers necrosis of cancer cells (Papo et al. 2004).

D-K_6_L_9_ is a molecule in which 1/3 of its natural aminoacid sequence has been replaced with diastereoisomers. Introduction of d aminoacids increases stability of this peptide in other organisms as it makes proteolysis more difficult and increases peptide selectivity (Papo et al. 2004). D-K_6_L_9_ features both hydrophobic aminoacids (leucine) and hydrophilic ones (lysine), which make it an amphipathic molecule. In aqueous solutions, this peptide does not preserve an ordered secondary structure, whereas after attachment to the lipid bilayer it adopts α-helical conformation (Papo and Shai 2003). It is preferentially bound by membranes rich in negatively charged acidic phospholipids (Papo et al. 2004, 2006), an augmented number of which are found in cancer cell membranes (Mader and Hoskin 2006). The peptide is bound by phosphatidylserine present in the outer membrane of the bilayer (Papo et al. 2006). The peptide is bound until it reaches threshold concentration upon which the membrane is depolarized and perforated causing cell death (Papo et al. 2004).

Therapeutic use of necrosis-triggering agents has its advantages and limitations. Among the most important factors released from necrotized cells is HMGB1 protein (Ellerman et al. 2007; Tang et al. 2011). Extracellular HMGB1 mediates chronic inflammatory condition, formation of new tumor microenvironment and angiogenesis. In other words, it facilitates tumor regrowth (Campana et al. 2008; Tang et al. 2010; Yang et al. 2005). On the other hand, inflammatory condition induced by HMGB1 and stimulation of antigen-presenting cell (APC) maturation can trigger immune response against cancer cells (Rovere-Querini et al. 2004; Zhu et al. 2010).

We tried to develop a combination therapy that would involve, besides D-K_6_L_9_, also other factors that would together totally destroy or inhibit B16-F10 melanoma tumors in experimental animals. Since surviving cancer cells and released HMGB1 protein can be responsible for tumor relapse, the proposed therapy involved factors that destroy the remnant cancer cells and that inhibit HMGB1.

HMGB1 inhibitors comprise specific antibodies, HMGB1 protein A box (Wang et al. 2012; Yang and Tracey 2010) or low-molecular compounds such as glycyrrhizin which inhibits HMGB1 following its release into extracellular matrix (Smolarczyk et al. 2012).

BP1 peptide binds to Flt-1 (VEGR1) receptor, thus blocking the attachment of vascular endothelial growth factor and placenta growth factor (PlGF). Inability of PlGF protein to bind to Flt-1 receptors found on the surface of cancer cells inhibits the latter’s motility and may affect the formation of metastases (Taylor and Goldenberg 2007). This peptide can also act effectively upon tumor endothelial cells possessing Flt-1 receptor (Tarallo et al. 2010). BP1 peptide has both anticancer properties (it inhibits proliferation of cancer cells) and is antiangiogenic.

Another therapeutic solution involves reinforcement of the immune system by applying interleukin (IL)-12. This cytokine activates secretion of interferon γ which, in turn, activates cytotoxic T lymphocytes and natural killer (NK) cells (Del Vecchio et al. 2007; Luedke et al. 2012; Uemura et al. 2010). Additionally, IL-12 inhibits angiogenesis, and formation of novel blood vessels (He et al. 2012; Rakhmilevich et al. 2004).

## Materials and Methods

### Plasmid pBCMGSNeo/IL-12

Plasmid pBCMGSNeo carrying a gene encoding murine IL-12 was obtained from Prof. H. Yamamoto (Osaka University, Japan). Murine IL-12 gene is composed of two subunits: p35 and p40 connected by cassette IRES. Gene of IL-12 cloned into BCMGSneo (so-called Karasuyama’s expression vector), under the control of cytomegalovirus. Plasmid preparations were isolated using QIAGEN-Endo Free Giga Kit (Qiagen GmbH, Hilden, Germany).

### Cell Culture

Murine melanoma B16-F10 and murine colon cancer C26 cells (ATCC) were propagated in RPMI 1640 supplemented with 10 % fetal bovine serum. Cell cultures were maintained in a standard 37 °C/5 % CO_2_ incubator. Cells were passaged every 2–3 days.

### Animals

Mice (6- to 8-week-old C57Bl/6 and BALB/c females) were from own animal facility. Consent for work with animals was obtained from the Local Ethics Commission (Silesian Medical University in Katowice).

### DNA Fragmentation Studies: TUNEL Test

The studies were accomplished with In Situ Cell Death Detection Kit, TMR red (Roche Diagnostic GmbH, Germany). The tests were performed using gelatin-coated 8-well Chamber Slide plates. Aliquots of 2 × 10^4^ B16-F10 cells per well were seeded in 250-μL culture medium. After 24 h, the medium was replaced with: 10, 20, 40 μg D-K_6_L_9_ peptide or fresh medium.

Following a 3-h incubation, the treated cells were fixed with 4 % paraformaldehyde. Next, the specimens were incubated on ice for 2 min with 0.1 % Triton X-100. Positive control cells were digested with 200 μL DNase solution (10 min at 37 °C). A mixture of fluorescein-labeled nucleotides and TdT enzyme was then added to the wells. After 1-h incubation at 37 °C, the specimens were embedded in glycerogel and observed using a Nikon Eclipse 80i fluorescence microscope (*λ* = 540 nm). Micrographs were taken using NISElements AR image analysis software.

### Hematoxylin and Eosin Staining

D-K_6_L_9_ peptide was injected intratumorally three times. Each time the treated mice received 100 μg D-K_6_L_9_ peptide in 100 μL physiological buffered saline (PBS^−^). Control mice were injected three times with PBS^−^ (100 μL per animal). Tumors were excised 24 h post-last injection. The collected material was fixed in 10 % formalin and embedded in paraffin. Five-μm-thick sections were then routinely stained with hematoxylin and eosin.

### Immunohistochemical Identification of HMGB1 Protein in B16-F10 and C26 Tumors Following D-K_6_L_9_ Peptide Administration

Tumors were excised 24 h following D-K_6_L_9_ peptide administration. The collected material was fixed with 10 % formalin, paraffin-embedded and cut into 5-μm-thick sections. The latter were deparaffinized and hydrated. Next, they were incubated with 0.3 % H_2_O_2_ and boiled with citrate buffer (10 mM; pH 6.0). Blocking of unspecific sites was achieved with 2.5 % horse serum. The sections were incubated for 1 h at RT using a primary rabbit anti-HMGB1 antibody [0.7 mg/mL; dilution 1:100; (Abcam, Cambridge, UK)] and with secondary horse anti-rabbit antibody conjugated to horseradish peroxidase (EC 1.11.1.7) from ImmPRESS^TM^ REAGENT Anti-Rabbit Ig kit (Vector, USA). Next, the specimens were incubated with diaminobenzidine (DAB) from ImmPACT^TM^ DAB kit. Brown-colored product was obtained as a result of enzymatic reaction. Finally, the specimens were dehydrated and were embedded using VectaMountTM.

### Animal Therapy Using D-K_6_L_9_ Peptide and Glycyrrhizin

C57Bl/6 mice were injected subcutaneously with 2 × 10^5^ B16-F10 melanoma cells. On the seventh and eighth day following inoculation, a 100 μg aliquot of D-K_6_L_9_ peptide was injected intratumorally (in 100 μL PBS^−^), whereas glycyrrhizin was injected intraperitoneally (2 mg/400 μL PBS^−^). For the subsequent 4 days, only glycyrrhizin was administered. The experiment was repeated twice.

### Animal Therapy Using D-K_6_L_9_ and BP1 Peptides

C57Bl/6 mice were injected subcutaneously with 2 × 10^5^ B16-F10 melanoma cells. On the seventh and eighth day following inoculation, a 100 μg aliquot of D-K_6_L_9_ peptide was injected intratumorally (in 100 μL PBS^−^). For the subsequent 3 days, BP1 peptide (in 100 μL H_2_O) was administered intratumorally. The experiment was repeated twice.

### Animal Therapy with D-K_6_L_9_ Peptide and pBCMGSNeo/IL-12

C57Bl/6 or BALB/c mice were injected subcutaneously with 2 × 10^5^ B16-F10 or C26 cells. On the seventh and eighth day following inoculation, a 100 μg aliquot of D-K_6_L_9_ peptide was injected intratumorally (in 100 μL PBS^−^). For the subsequent 9 or 10 days pBCMGSNeo/IL-12 only was administered intratumorally (50 μg in 100 μL PBS^−^). Control group mice received only plasmid DNA BCMGSNeo (empty plasmid), pBCMGSNeo/IL-12, D-K_6_L_9_ peptide, pBCMGSNeo (empty plasmid) with D-K_6_L_9_ peptide or PBS^−^ (doses and timing as in experimental groups). Plasmid DNA BCMGSNeo is the carrier of the therapeutic gene IL-12. To exclude the therapeutic effect of empty vector, pBCMGSNeo created two groups of control mice treated with: a combination of peptide and pBCMGSNeo (empty vector) and alone pBCMGSNeo (empty vector). The experiment was repeated three times.

### Determination of CD8^+^, CD4^+^ and NK Cell Numbers in B16-F10 Melanoma Tumors During Therapy with D-K_6_L_9_ Peptide and pBCMGSNeo/IL-12

B16-F10 tumor material was collected for FACS analysis and single-cell suspension obtained with digestion mixture [0.5 mg/mL collagenase A, (Sigma Aldrich, MO, USA); 0.2 mg/mL hyaluronidase type V, (Sigma Aldrich, MO, USA); 0.02 mg/mL DNase I, (Roche Diagnostic GmbH, Germany); per each 0.25 g of tumor tissue). Red blood cells were lysed using 0.15 M ammonium chloride solution (Sigma Aldrich, MO, USA)]. Dead cells were removed by centrifugation on Lympholyte-M gradients (Cedarlane, Canada). Level of T lymphocytes was determined in homogenous single-cell suspension. To identify the subpopulations of T lymphocytes, the following antibodies were used: PE-Cy™7-CD3e, PE-CD4 and FITC-CD8a (BD, Franklin Lakes, NJ, USA). NK cells were identified with an anti-mouse CD49b antibody (Biosciences, CA, USA). Gate parameters dividing negative from positive cells were chosen based on isotype antibody control probes (Jarosz et al. 2013).

### Statistical Analysis

Differences between groups were determined by applying ANOVA followed by the Tukey’s post hoc test. A *p* value lower than 0.05 was considered statistically significant.

## Results

### D-K_6_L_9_ Peptide Does Not Cause DNA Fragmentation (TUNEL Test)

**Fig. 1:**
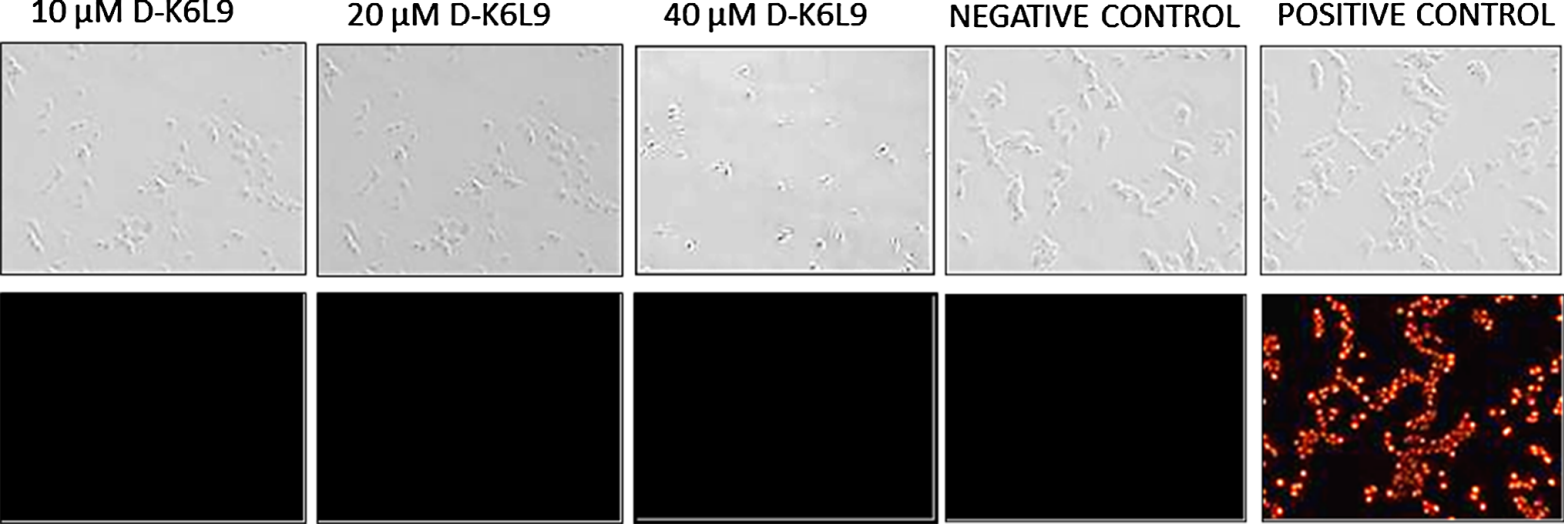
Identification of DNA fragmentation using TUNEL test. Test was accomplished using three parallel cell cultures: untreated cells (negative control), DNase-treated cells (positive control), peptide-treated cells (10, 20, 40 μM D-K_6_L_9_). Red fluorescence reflecting DNA fragmentation was found only in positive control. Objective lens magnification ×10

TUNEL test was performed on B16-F10 melanoma cells subjected to 3-h incubation with three different concentrations of D-K_6_L_9_ peptide (10, 20 or 40 μM), DNase (positive control) and on untreated cells (negative control). Positive control cells showed intense red fluorescence, thus confirming DNA fragmentation. Cells treated with D-K_6_L_9_ peptide, similarly as negative control cells, did not display fluorescence. This shows that the examined peptide does not cause fragmentation at the times that have been studied (Fig. 1).

### D-K_6_L_9_ Peptide Increases Necrotic Regions in B16-F10 and C26 Tumors and Triggers HMGB1 Release

**Fig. 2:**
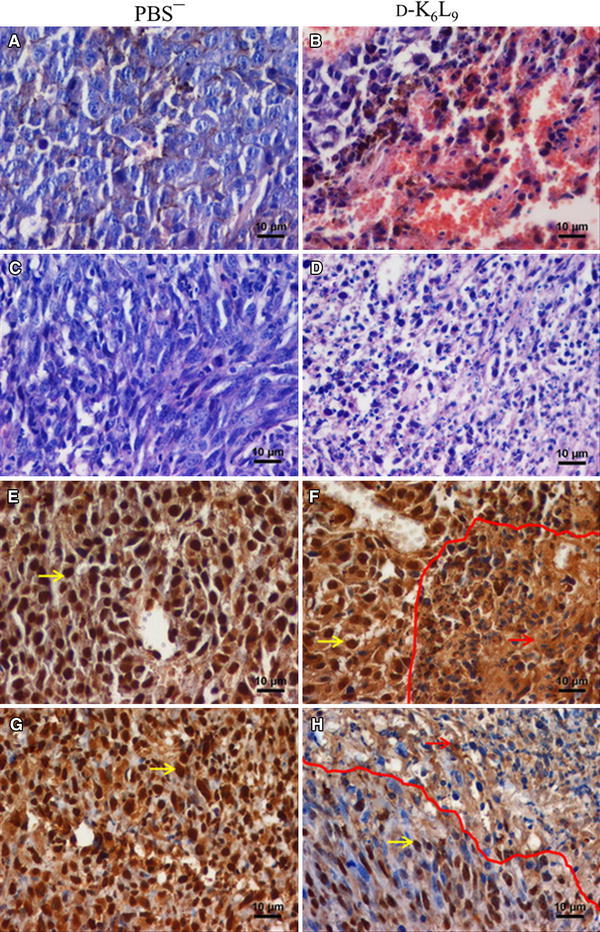
Immunohistochemical assessment of B16-F10 and C26 tumors following therapy with D-K_6_L_9_ peptide. Micrographs shows staining of control tumors **a**, **c**, **e**, **g** (injected with PBS^−^) and **b**, **d**, **f**, **h** tumors injected with D-K_6_L_9_ peptide. Hematoxylin and eosin staining: **a**, **b** B16-F10 and **c**, **d** C26. Necrotic areas visible in sections derived from tumors treated with the peptide. Objective lens magnification ×10. Identification of HMGB1: **e**, **f** B16-F10 and **g**, **h** C26. *Brown-colored* staining represents HMGB1 protein, cell nuclei are stained *blue* (hematoxylin). In control tumors, HMGB1 protein is present mainly in cell nuclei (*yellow arrows*). In tumors injected with D-K_6_L_9_ peptide, HMGB1 is present outside of cell nuclei and in extracellular space (*red arrows*). *Red line* indicates the border between the area of necrosis and the remaining tissue area. Objective lens magnification ×40

Sections from D-K_6_L_9_ peptide-treated tumors were stained to show HMGB1 protein presence. As a result of treatment with this peptide, HMGB1 was released into cytoplasm, unlike in controls where the protein is confined to cell nucleus (Fig. 2).

### D-K_6_L_9_ Peptide and Glycyrrhizin Combination Inhibits Growth of B16-F10 Melanoma Tumors but Does Not Prolong Animal Survival

**Fig. 3:**
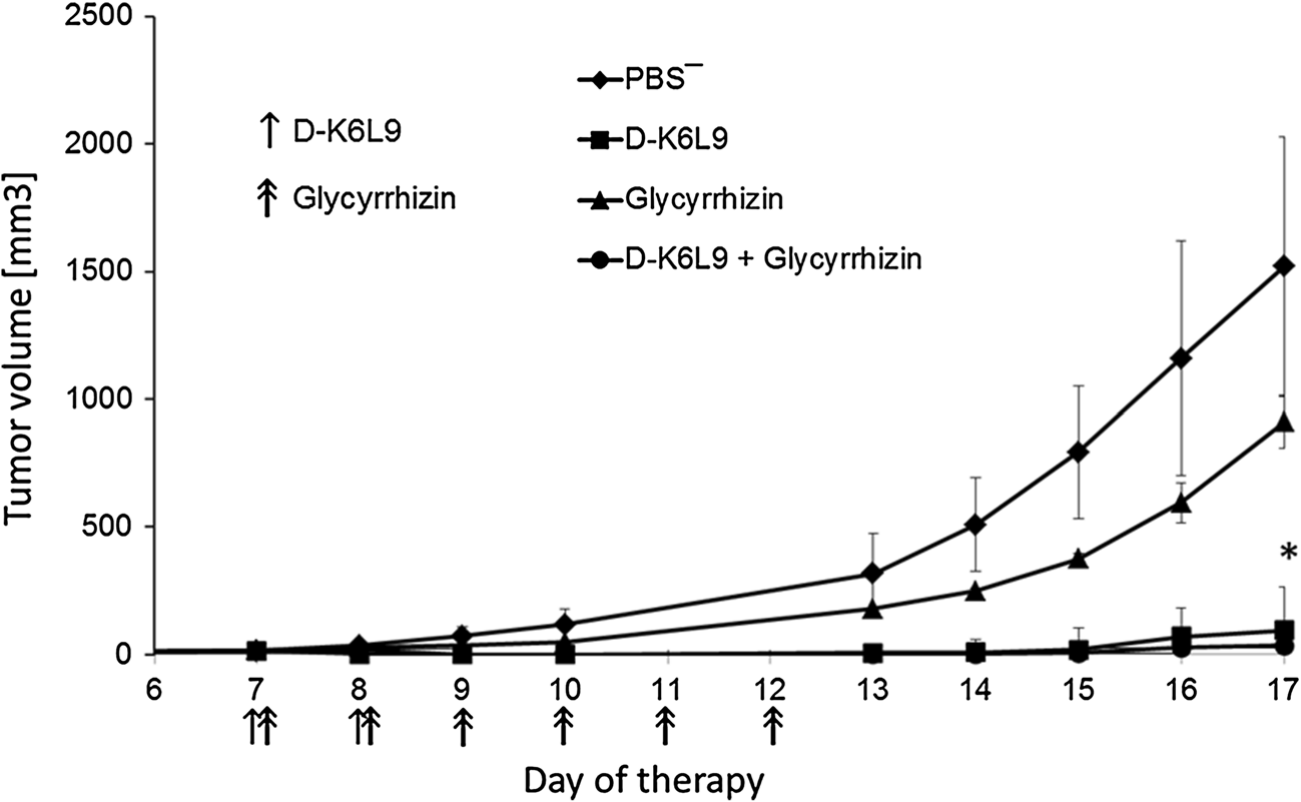
Effect of D-K_6_L_9_ peptide combination with glycyrrhizin on growth of B16-F10 murine melanoma tumors. On days 7 and 8 following inoculation of mice with B16-F10 cells, the animals were injected intratumorally with D-K_6_L_9_ peptide (100 μg/100 μL) and intraperitoneally with 2 mg glycyrrhizin. Next, for four consecutive days only glycyrrhizin was administered. Animals in control groups received D-K_6_L_9_ peptide, glycyrrhizin and PBS^−^ in same time sequences as those treated with combination therapy. Each group consisted of five animals. Differences in tumor volumes between mice that received D-K_6_L_9_ peptide and mice that received peptide and glycyrrhizin were statistically significant (*p < 0.009 Tukey post hoc analysis after ANOVA) on day 17 of the therapy

D-K_6_L_9_ peptide combined with glycyrrhizin inhibits the growth of B16-F10 melanoma tumors as opposed to control groups of animals treated with peptide or glycyrrhizin only (Fig. 3). After cessation of therapy, tumor growth was resumed. Inhibition of tumor growth did not extend survival of the treated animals (data not shown).

### D-K_6_L_9_ and BP1 Peptide Combination Inhibits Growth of B16-F10 Melanoma Tumors but Does Not Prolong Animal Survival

**Fig. 4:**
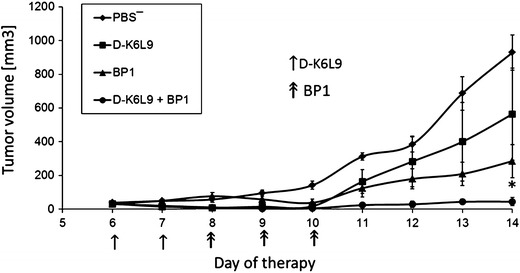
Effect of D-K_6_L_9_ peptide combination with BP1 peptide on growth of B16-F10 murine melanoma tumors. On days 7 and 8 following inoculation of mice with B16-F10 cells, the animals were injected intratumorally with D-K_6_L_9_ peptide (100 μg/100 μL). Next, for three consecutive days only BP1 peptide (100 μg/100 μL) was administered. Animals in control groups received D-K_6_L_9_ peptide, BP1 peptide and PBS^−^ in same time sequences as those treated with combination therapy. Each group consisted of five animals. Differences in tumor volumes between mice that received D-K_6_L_9_ peptide or BP1 peptide and mice that received both peptides were statistically significant (*p < 0.02 Tukey post hoc analysis after ANOVA) on day 14 of the therapy

D-K_6_L_9_ and BP1 peptide combination inhibits the growth of B16-F10 melanoma tumors as opposed to control groups of animals treated only with either peptide (Fig. 4). After cessation of therapy, tumor growth was resumed. Inhibition of tumor growth did not extend survival of the treated animals (data not shown).

### D-K_6_L_9_ Peptide Combined with IL-12 Inhibits Growth of B16-F10 Melanoma and C26 Murine Colon Carcinoma Tumors and Prolongs Animal Survival

**Fig. 5:**
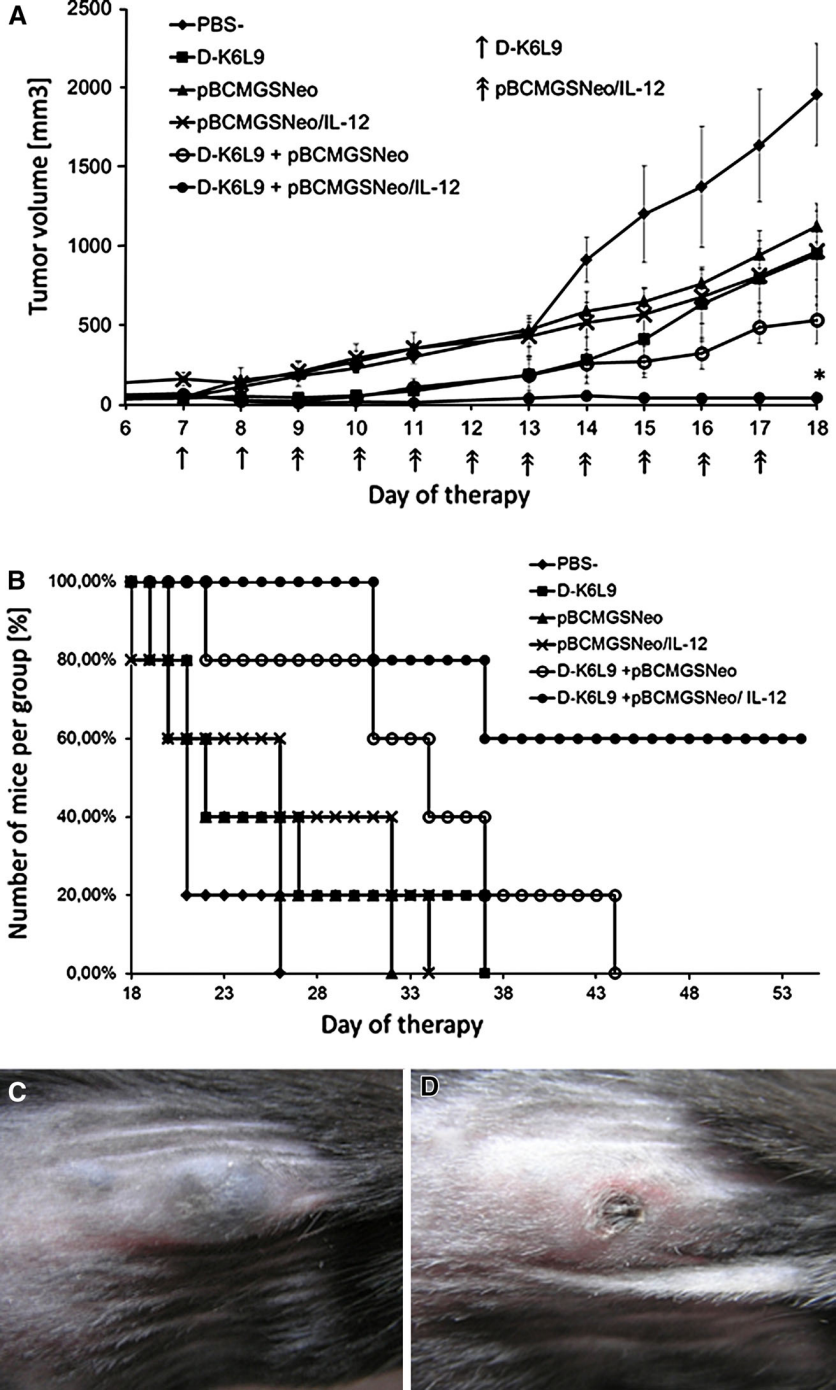
Effect of D-K_6_L_9_ peptide combination with IL-12 on growth of B16-F10 murine melanoma tumors and survival of the treated mice. On days 7 and 8 following inoculation of mice with B16-F10 cells, the animals were injected intratumorally with D-K_6_L_9_ peptide (100 μg/100 μL). Next, for nine consecutive days, pBCMGSNeo plasmid (50 μg/100 μL PBS^−^) carrying IL-12 gene was administered intratumorally. Mice in control groups received pBCMGSNeo (empty vector), pBCMGSNeo (empty plasmid) with D-K_6_L_9_, pBCMGSNeo/IL-12, D-K_6_L_9_ peptide or PBS^−^ in same time sequences as those treated with combination therapy. Each group consisted of five animals. **a** Combined therapy D-K_6_L_9_ + pBCMGSNeo/IL-12; tumor growth vs. time. **b** Combined therapy D-K_6_L_9_ + pBCMGSNeo/IL-12; animal survival. *p < 0.003 Tukey post hoc analysis after ANOVA. Images of tumors were performed in the 13th day of therapy. **c** Control group D-K_6_L_9_; **d** combined therapy D-K_6_L_9_ + pBCMGSNeo/IL-12

**Fig. 6:**
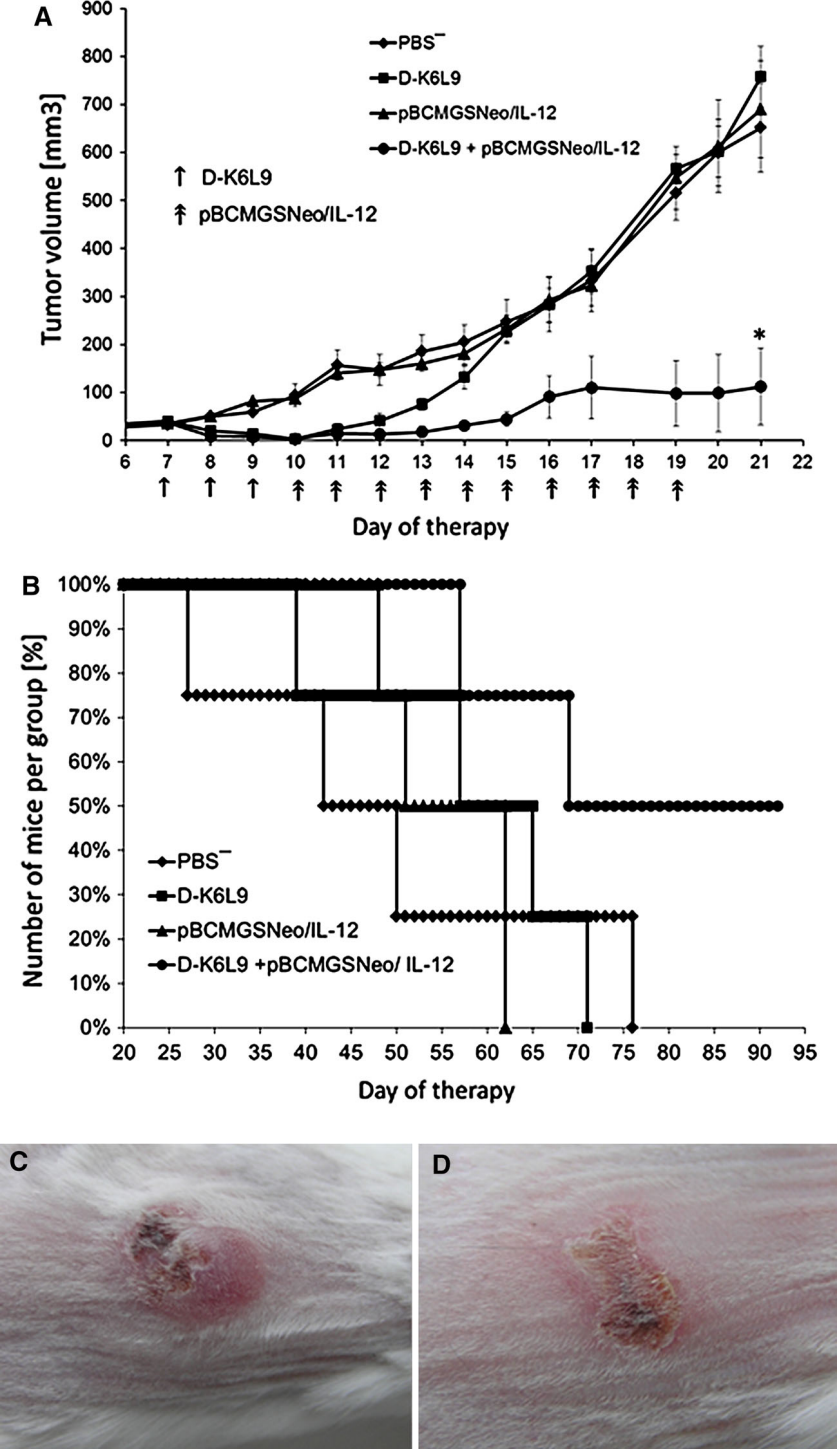
Effect of D-K_6_L_9_ peptide combination with IL-12 on growth of C26 colon carcinoma tumors and survival of the treated mice. On days 7 and 8 following inoculation of mice with C26 cells, the animals were injected intratumorally with D-K_6_L_9_ peptide (100 μg/100 μL). Next, for 10 consecutive days, pBCMGSNeo plasmid (50 μg/100 μL PBS^−^) carrying IL-12 gene was administered intratumorally. Mice in control groups received pBCMGSNeo/IL-12, D-K_6_L_9_ peptide or PBS^−^ in same time sequences as those treated with combination therapy. Each group consisted of five animals. **a** Combined therapy D-K_6_L_9_ + pBCMGSNeo/IL-12; tumor growth vs. time. **b** Combined therapy D-K_6_L_9_ + pBCMGSNeo/IL-12; animal survival. *p < 0.002 Tukey post hoc analysis after ANOVA. Images of tumors were performed in the 13th day of therapy. **c** Control group D-K_6_L_9_; **d** combined therapy D-K_6_L_9_ + pBCMGSNeo/IL-12

D-K_6_L_9_ combined with pBCMGSNeo/IL-12 does inhibit the growth of B16-10 melanoma tumors as compared to control mice treated with PBS^−^, pBCMGSNeo (empty vector) pBCMGSNeo/IL-12, pBCMGSNeo (empty plasmid) with D-K_6_L_9_ peptide or D-K_6_L_9_ only (Fig. 5a). In 60 % of cases, significant extension of survival period for combination treatment was observed (Fig. 5b). The increase in survival time of mice was associated with a complete loss of primary tumors (Fig. 5d). Similar effect was observed for C26 murine colon carcinoma tumors (Fig. 6a). In 50 % of cases, significant extension of survival period for combination treatment was observed (Fig. 6b). The increase in survival time of mice was associated with a complete loss of primary tumors (Fig. 6d). This effect was observed even after 2 months following conclusion of therapy; mice were alive without any visible foci of B16-F10 melanoma tumors.

### Combination of D-K_6_L_9_ Peptide with pBCMGSNeo/IL-12 Causes Increase in the Number of CD8^+^, CD4^+^ and NK Cells in B16-F10 Tumors

**Fig. 7:**
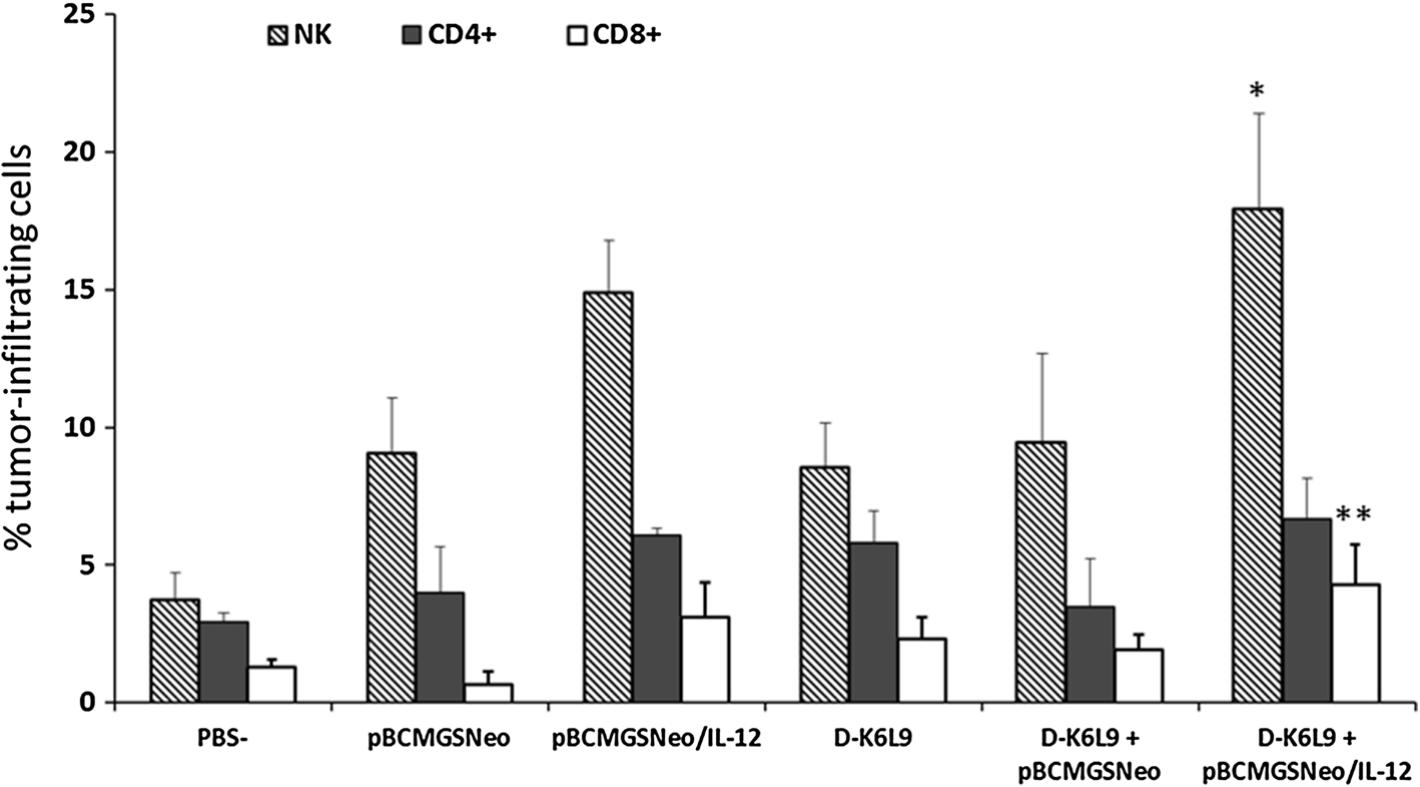
Level of T lymphocytes and NK cells in B16-F10 murine melanoma tumors treated with combination of D-K_6_L_9_ peptide and IL-12. Analyzed tumor material was collected 24 h from last administration of treatment agent (PBS^−^, pBCMGSNeo (empty plasmid), pBCMGSNeo (empty plasmid) with D-K_6_L_9_, pBCMGSNeo/IL-12 or D-K_6_L_9_). PBS^−^ denotes two administrations of PBS^−^; D-K_6_L_9_—two administrations of peptide; pBCMGSNeo—six administrations of empty vector; pBCMGSNeo/IL-12—six administrations of plasmid DNA; D-K_6_L_9_ + pBCMGSNeo/IL-12—two administrations of peptide and six administrations of pBCMGSNeo/IL-12 plasmid DNA. *Bars* represent averages 7–8 tumors. ANOVA: *p < 0.027 D-K_6_L_9_ + pBCMGSNeo/IL-12/D-K_6_L_9_ + pBCMGSNeo; **p < 0.049 D-K_6_L_9_ + pBCMGSNeo/IL-12/D-K_6_L_9_ + pBCMGSNeo

Mice treated with D-K_6_L_9_ peptide and pBCMGSNeo/IL-12 combination showed a significantly increased levels of tumor infiltrating CD8^+^ T lymphocytes and NK cells (Fig. 7). However, increases in the number of infiltrating cells CD4^+^ were not observed (Fig. 7). Animals treated with pBCMGSNeo (empty vector), pBCMGSNeo (empty plasmid) with D-K_6_L_9_, pBCMGSNeo/IL-12, peptide alone also showed increased numbers of T lymphocytes and NK cells but not to the same degree as in mice subjected to combinatory treatment. The latter clearly activates both specific and nonspecific immune response.

## Discussion

Peptides are among agents intensively investigated for anticancer properties. They show several advantages, such as ease of synthesis, high degree of tumor tissues penetration or low immunogenicity. Among disadvantages is their relatively short half-time in circulation (Smolarczyk et al. 2009).

A synthetic D-K_6_L_9_ peptide selectively destroys cancer cells by damaging membrane structures (Papo et al. 2003). This leads to necrotic type of cell death (Papo et al. 2004).

Such a mechanism of cell death following exposure to this peptide can be inferred from lack of DNA fragmentation or lack of active caspase 3 (unpublished results), which confirms observations made by Papo et al. (2004).

In our experiments involving B16-F10 murine melanoma and C26 colon carcinoma tumors, we observed inhibited tumor growth (compared to control) only during therapy with the peptide. Papo et al. (2004, 2006) showed inhibitory effect of the peptide using 22RV1 and CL1 prostate tumor, as well as MDA-MB-231 breast tumor models.

Following D-K_6_L_9_ peptide administration, we observed necrosis in tumor tissue as well as release of HMGB1 protein into extracellular space. The released HMGB1 activates division of surviving cancer cells, maintains inflammatory condition, stimulates the formation of tumor microenvironment towards angiogenesis. It thus contributes to sustained tumor growth (Smolarczyk et al. 2012; Tang et al. 2010). On the other hand, inflammatory condition induced by HMGB1 and stimulation of APC can be of significance for triggering specific immune response directed against cancer cells (Zhu et al. 2010). Numerous data have shown that tumor relapse is mediated by surviving cancer cells or cancer stem cells (CSC) (Baguley 2006; Diaz and Leon 2011). Despite the fact that CSCs constitute only a small percent of all cancer cells in a tumor, they are CSCs that are responsible for tumor chemo- and radioresistance (Bourguignon et al. 2008; Diaz and Leon 2011; Diehn et al. 2009; Holtz et al. 2005; Nandi et al. 2008; Shafee et al. 2008). This is explained by great resistance to apoptosis, elevated levels of repair proteins and ABC-type proteins mediating removal of toxins (Eyler and Rich 2008; Hirschmann-Jax et al. 2004).

It has been believed that effective therapy must lead to complete eradication of CSC (Blagosklonny 2005; Bonavia et al. 2011; Tang 2012). To achieve this, drugs are used that act independently of cell cycle (Diehn et al. 2009; Massard et al. 2006; Morrison et al. 2011), along with ABC protein inhibitors (Dean et al. 2005; Dylla et al. 2008; Ginestier et al. 2007) and immunotherapy (Cioffi et al. 2012; Lai et al. 2012; Ning et al. 2012; Parmiani et al. 2007; Pietra et al. 2009). A more promising strategy, however, consists of acting upon CSC niche which includes endothelial cells forming tumor blood vasculature, macrophages, fibroblasts and immune system cells (Baguley 2006; Calabrese et al. 2007). To inhibit tumor relapse, two solutions are possible. First involves blocking HMGB1 activity which is conducive towards tumor growth. Second solution consists of enhancing immune system response directed against therapy-surviving cancer cells and CSC.

Our study was an attempt to develop a combination therapy involving D-K_6_L_9_ peptide and agents that might strengthen its action. To achieve this, we used the following drugs: glycyrrhizin (an inhibitor of HMGB1), BP1 peptide (angiogenesis inhibitor) and IL-12 (a cytokine having immunostimulatory properties).

Glycyrrhizin administered intraperitoneally to mice previously treated with a peptide only inhibited growth of B16-F10 melanoma and C26 colon carcinoma tumors. A similar effect was observed when using a combination of glycyrrhizin with CAMEL peptide, which also triggers cell necrosis (Smolarczyk et al. 2010, 2012). In most likelihood tumor relapse that we observed following cessation of therapy was mediated by surviving cancer cells or CSC. Combination of glycyrrhizin with D-K_6_L_9_, as with CAMEL, is not very efficient. Irrespective of peptide used to induce necrosis, blocking HMGB1 only proved insufficient. To totally inhibit tumor growth, it is necessary to eliminate the remaining surviving cancer cells.

We further tested BP1 peptide which has both anticancer properties (it inhibits growth of cancer cells) and acts as an antiangiogenic (by inhibiting angiogenesis activated by HMGB1). In our opinion, due to its dual mode of action, this peptide matched very well the assumptions behind our combination strategy with D-K_6_L_9_. We observed inhibition of tumor growth only during the treatment, after which tumors invariantly regrew. Similarly, like in the case of glycyrrhizin, surviving cancer cells or CSCs, not reached by BP1, are responsible. Since both therapies aimed at inhibiting activity of HMGB1 proved not very effective, we decided to check the combination involving D-K_6_L_9_ peptide and IL-12. The latter cytokine activates T lymphocytes and NK cells which infiltrate tumor mass and tissues in its vicinity and destroy cancer cells (Del Vecchio et al. 2007; Uemura et al. 2010).

Combination of D-K_6_L_9_ and IL-12 inhibits tumor growth and extends survival of the treated animals. In ca. 60 % of mice that harbored B16-F10 melanoma tumors and in 75 % of mice with C26 colon carcinoma tumors, total disappearance of tumors was observed.

A similar effect was observed when combining CAMEL peptide and plasmid DNA carrying IL-12 gene, where tumors disappeared in 60 % of mice bearing B16-F10 melanoma tumors (Smolarczyk et al. 2010). IL-12 combination with drugs that cause necrotic tumor cell death (CAMEL, D-K_6_L_9_) seems to be the best therapeutic option. IL-12 enhances response of the immune system, and activates NK cells to destroy residual tumor cells after treatment which are responsible for tumor re-growth. In mice that were treated with combination therapy involving D-K_6_L_9_ and IL-12, infiltration of CD4^+^, CD8^+^ and NK cells was confirmed. Combination peptide with BP1 or glycyrrhizin only inhibits tumor regrowth by inhibiting angiogenesis. This is the reason why only temporary inhibition of tumor growth is observed after cessation of therapy. Cancer cells remaining alive after treatment with the peptide are responsible for this growth and are resistant to anti-angiogenic therapy. Therapeutic approach involving the use of anti-angiogenic therapy after radically destroying the tumor is ineffective.

IL-12 has pleiotropic properties: activates specific and non-specific response of the immune system but also has anti-angiogenic properties (Kilinc et al. 2006; Nanni et al. 1998). The use of IL-12 in combination with peptide not only inhibits angiogenesis but most importantly destroys survivors' cancer cells, responsible for tumor regrowth. Effective cancer therapy should be based on a number of therapeutic solutions, for example, anti-angiogenic or antivascular therapy, but should essentially include immunotherapy.

To conclude, a therapeutic strategy consisting of joined administration of D-K_6_L_9_ peptide that induces necrosis of cancer cells with a cytokine activating immune system cells (IL-12) proved highly effective in inhibiting growth of two types of experimental tumors.

## Abbreviations

D-K_6_L_9_: Peptide-Ac[D(K_6_L_9_)]NH_2_
BP1: Peptide-(SHRYRLAIQLHASDSSSSCV)
B16-F10: Murine melanoma cells
C26: Colon carcinoma cell
CSC: Cancer stem cells
HMGB1: High-mobility group box-1 protein
NK: Natural killer cells
VEGR: Vascular-endothelial growth receptor
PlGF: Placenta growth factor
IL-12: Interleukin-12
PBS^−^: Physiological buffered saline
RPMI 1640: Cell culture medium
